# Clean Milk

**Published:** 1916-04

**Authors:** Ethel Lyon Heard

**Affiliations:** Galveston, Texas


### Clean Milk.

ETHEL LYON HEARD, GALVESTON, TEXAS.

The gradual change in economic conditions which has developed all over the country in the past quarter or half a century has affected the family supply of dairy products perhaps more deleteriously than any other one article or group of articles used or consumed by the individual members. Time was when nearly every family had a cow or cows, and clean butter and milk was a matter of course. In smaller towns, where the milkman peddled his product, conditions were perhaps less desirable, but the comparatively short time which elapsed between the milking and the delivery and use of the milk materially tended toward its better condition. With the growth of cities, the consequent recession of the origin of the supply of milk from its market, and the commercial aspect of that marketing as well as its production, have both tended strongly to deteriorate the quality of this perishable food, and bring it to the consumer in a condition all too frequently actively dangerous if used raw, and unfit for small children and infants, raw or cooked.

A good many cities have awakened to the necessity of strict regulation of their milk supply. The urgency of the question is evinced by the existence of the New York Milk Committee, the American Association of Milk Commissions, and other bodies of persons interested in and alive to the necessity of pure milk, and of practical methods for securing it. Furthermore, at the present time, there is a resolution pending in the House of Representatives at Washington calling for the thorough investigation of dairies and dairy products by a Federal commission, owing to the fact that said dairies and dairy products are almost universally of such a nature as to be a serious menace to the health and welfare of the people.

It is a fact too well known to require discussion here, that the organisms of almost any infectious disease may be conveyed and transmitted by a contaminated milk supply. Typhoid fever, diphtheria and septic sore throat are notable instances, and outbreaks of scarlet fever have been traced to milk supplies. Tuberculous cows in a herd offer a constant menace to the consumers of their milk, and even in the absence of actual known pathogenic bacteria the swarms of organisms that are present in milk, which has been drawn into unclean vessels by dirty hands, from a dirty cow in a dirty stable, improperly cooled or not cooled at all, and marketed when twelve to twenty-four hours old, are sufficient in themselves to precipitate the most severe gastro-enteritis or ileocolitis in infants, and even when such milk is pasteurized the dead bodies of these myriad organisms contain sufficient toxins to upset the delicate digestive apparatus of a baby.

For a wholesome milk supply, which we will therefore concede is a necessity in any community, certain things are necessary. Not elaborate barns with concrete floors, patent yokes and expensive sterilizers and pasteurizers, and bottlers and clarifiers, and all the other money dissipating machinery that has been devised to beguile the unwary farmer. Three things will insure clean, safe milk.

First, inspection of the herd by a competent veterinarian at least twice a year, and the prompt elimination of all tuberculous or other diseased cows.

Second, *cleanliness*. Clean milk pails that have been *scalded* before use, with small tops and *no strainers*. It's far more sensible to keep dirt out of the milk than it is to try to strain it out after the damage of contamination is already done. A milker who has washed and dried his hands, and has on clean clothes, a cow whose flank and udder are free from long hair, dust, mud and manure, and have been wiped with a moist cloth just previous to milking, a clean stable free from flies and dust. Clean, *scalded* cans or other containers to receive the milk.

Third, *temperature*. Immediately after milking the temperature of the milk should be reduced to 55° F. or below and *kept there until the milk reaches the consumer*. This may be accomplished by setting the cans in ice or cold well or spring water, the latter being allowed to flow around the cans until the proper degree of coolness is obtained.

What steps shall a community take to insure for itself clean milk? This is a question more complex and difficult to answer the larger the community and the more numerous its sources of supply. A milk ordinance covering the inspection of herds, condition of stables and premises, temperature of milk at the time it is offered for sale, and providing for adequate laboratory examination of samples may be worked out for each community by persons interested in correcting local conditions. A careful study of Bulletin No. 85, issued by the Public Health and Marine Hospital Service, 'Methods and Standards for the Production and Distribution of Certified Milk"; also No. 78, "Report of the Commission on Milk Standards Appointed by the New York Milk Committee," will be of great help. A good inspector who can gain the confidence of the dairy men and interest them in improving their product is of the greatest value. But after all, the strongest factor in bringing about any reform is public opinion. If each housewife could be informed of the nature and condition of her milk supply the demand for clean milk or no milk would speedily induce the dealers to remedy their deficiencies.

A word about the importance of laboratory examination of milk. This fluid being physically constituted as it is, it is not possible for the individual consumer to know or ascertain the exact condition of her milk supply. While the nutritive value of milk may be greatly decreased by the removal of the cream, or diluting with various proportions of water, and while both these procedures are violations of the State Pure Food Law, these conditions in a milk supply are not the flagrant danger that an enormous bacterial content is, or the presence of the bacteria of disease. The housewife may suspect that her milk is watered or skimmed, but there is no way in the world for her to know whether she is feeding her baby tubercle bacilli, typhoid organisms or a mass of saprophytes so enormous as to be in itself a menace. The only defense the mothers of a community have against this actual danger is the alertness of the governing authority of that community to safeguard it from such conditions. And the only criterion by which the governing authority can be guided, apart from inspection of actual conditions at the dairies, is the result of the laboratory examinations of samples of milk offered for sale by these dairies. I have known a medical man, presumably a well educated and competent person, to declare before the governing body of a city endeavoring to improve its milk supply, that "bacterial counts on milk were all nonsense; that *nobody* could count a million bacteria." In spite of the attitude of this man, it is, nevertheless, true that a fairly accurate knowledge of the average condition of the milk of a given dairy can be arrived at by five or six successive laboratory examinations of that milk, including the bacterial count, and a continued high count, persisting in spite of efforts to induce the producer to clean up his dairy and his methods, should be a sufficient reason for restraining him from selling his milk.

The welfare of a community should be the individual care of each member of that community. If you know there is an abuse in existence, *do* something about it; don't close your eyes and let it go. The women are a strong factor for improvement if they can be interested in a question, and every woman surely should be interested in safe food for her children.

